# Supplementary material for: SERS discrimination of single DNA bases in single oligonucleotides by electro-plasmonic trapping
Source: Nat Commun. 2019 Nov 22;10:5321. doi: 10.1038/s41467-019-13242-x (PMC6874578; doi:10.1038/s41467-019-13242-x)
Supplement: Supplementary file 1 — Supplementary Information [file 41467_2019_13242_MOESM1_ESM.pdf]

Supplementary Information

SERS Discrimination of Single DNA Bases in Single Oligonucleotides by Electro-plasmonic Trapping

Huang et al.

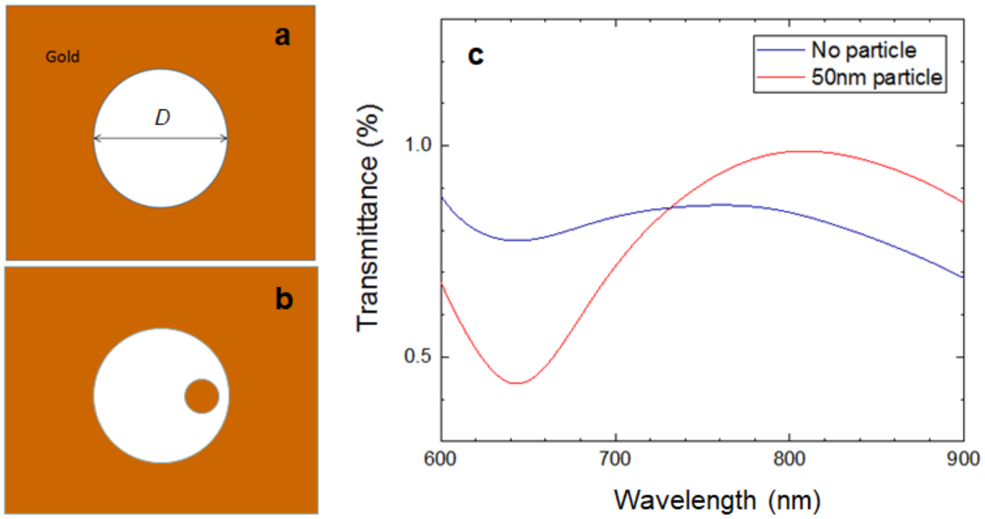

Supplementary Figure 1. Nanoparticle-nanohole coupling. Simulation model of (a) a gold nanohole with a diameter, *D*, and (b) a ϕ50 nm gold nanoparticle in the nanohole. (c) Simulated far-field transmission of the *D* = 200 nm gold nanohole with and without a ϕ50 nm gold nanoparticle in it.

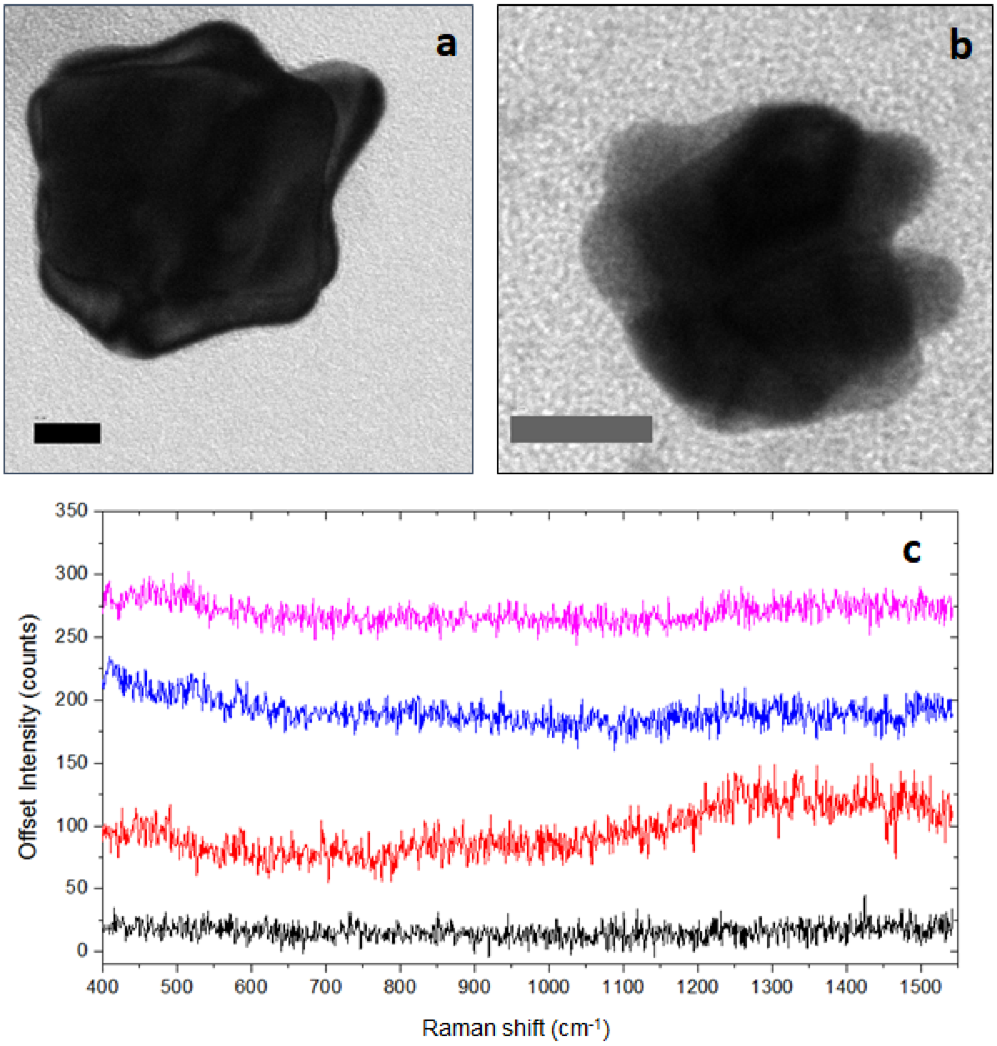

Supplementary Figure 2. Morphology and Raman signals of non-functionalized gold nanourchin (AuNU). (a,b) TEM images of the AuNUs. The scale bar in (a) is 10 nm and the scale bar in (b) is 20 nm. (c) Measured Raman spectra of the unmodified AuNUs diffusion in solution, in which the black curve was the background of the solution. They may originate from the citrates that were loosely adsorbed on the AuNUs for stabilization.

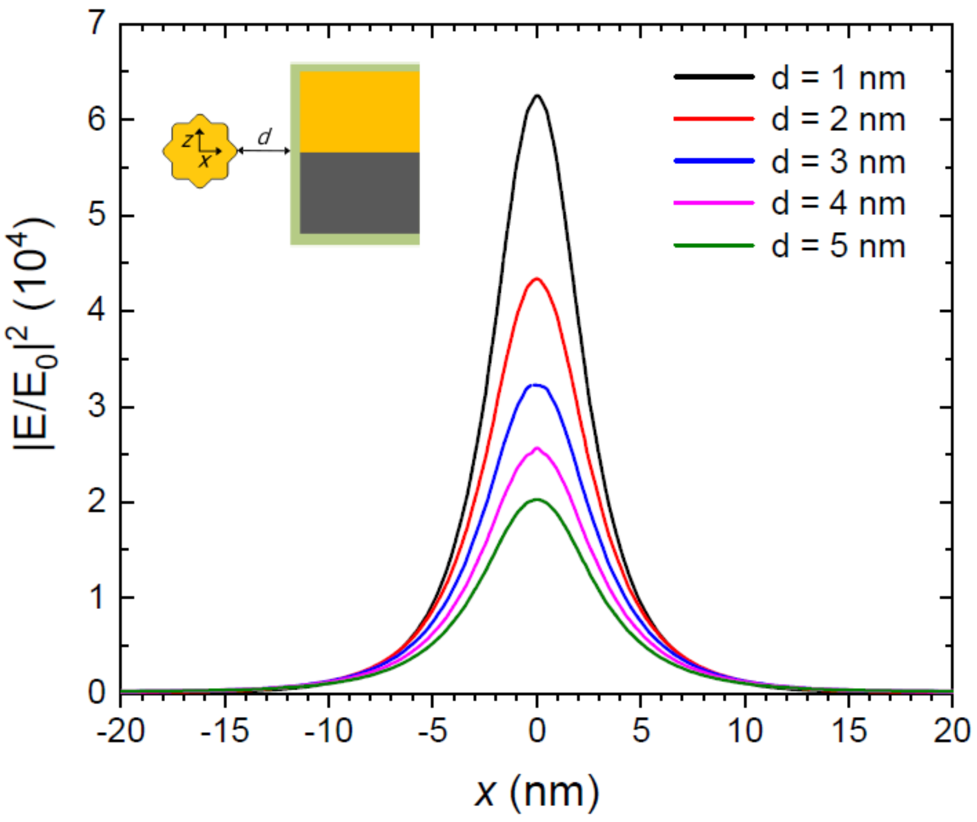

Supplementary Figure 3. Distance-dependent field enhancement and confinement. Simulated field intensity enhancement and distribution on the AuNU tip with different gap distance (*d*) between the AuNU tip and the alumina layer coated on the nanohole suggests that smaller gap leads to stronger and more confined electromagnetic fields. Inset shows the simulated model with coordinates.

Supplementary Note 1. Optical forces and Brownian motion

The first condition for optical trapping is that the backward axial gradient force (F_{grad}) should be larger or equal to the forward scattering force (F_{scat}):[1, 2]

$$\frac{F_{\text{grad}}}{F_{\text{scat}}} \geqslant 1 \tag{1}$$

Supplementary Figure 4 below shows that the F_{scat} reaches a maximum of 0.58 pN in the -z direction at 5 nm distance between the AuNU and the nanohole sidewall, which is 5 times lower than F_{grad} (3 pN) in the x direction on Figure 2b in the manuscript.

The second condition for optical trapping requires that the potential energy of the gradient optical force should be much larger than the kinetic energy of the Brownian motion:[2]

$$\exp\left(\frac{U}{k_b T}\right) \gg 1 \tag{2}$$

where $U = \frac{4n_2R^3}{c}\left(\frac{\varepsilon_1-\varepsilon_2}{\varepsilon_1+2\varepsilon_2}\right)\frac{P}{w_0^2}$ is the potential energy of the gradient optical force, in which k_b is the Boltzmann constant, T is the temperature of the environment around nanoparticle, n_2 is the refractive index of the environment, R is the radius of nanoparticle, c is the speed of light, ε_1 and ε_2 are the dielectric constants of the nanoparticle and environment respectively, P is the laser power, and w_0 is the waist radius of the laser beam. In our case of AuNUs with diameters of 50 - 60 nm and 10 mW laser power, the expression $\exp\left(\frac{U}{k_b T}\right)$ approximately equals from 5 to 17, which confirms the dominance of the optical force over the Brownian motion.

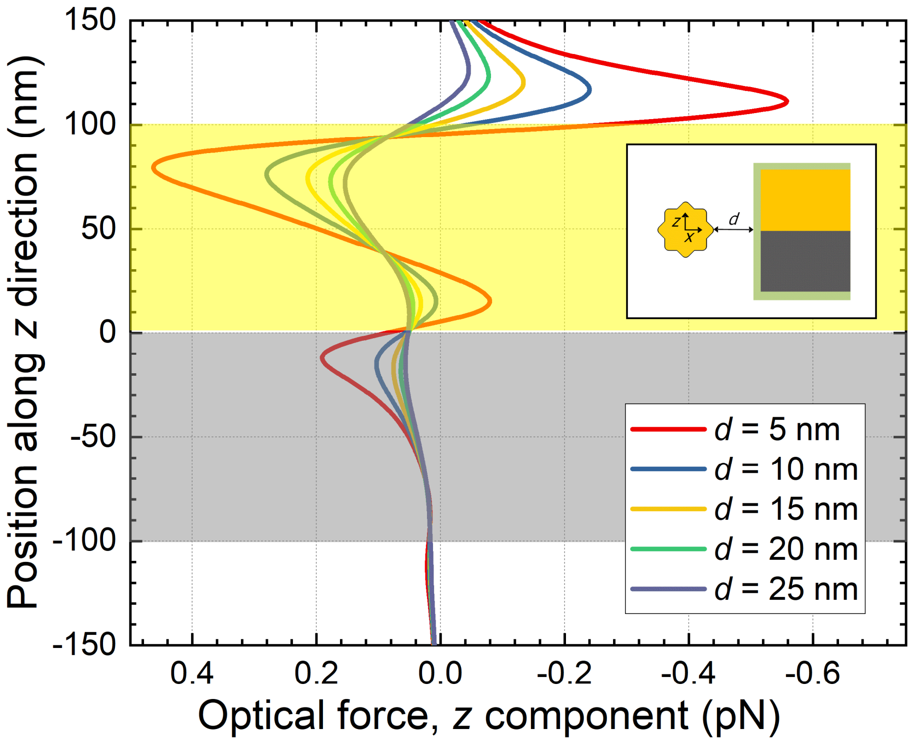

Supplementary Figure 4. Distance-dependent optical scattering force. Simulated *z* component of optical scattering force (F_{scat}) under 10 mW laser of 785 nm wavelength changes with varying gold nanourchin (AuNU) position along the *z* direction. *d* is the distance between the AuNU tip and the nanohole sidewall. Inset shows the simulated model with coordinates.

Supplementary Table 1. Measured Zeta potentials (ζ_{np}) of non-functionalized, multilayer and sub-monolayer nucleobase-AuNUs solutions.

		**ζ_{np} 1 (mV)**	**ζ_{np} 2 (mV)**	**ζ_{np} 3 (mV)**
**Non-functionalized**	**AuNU**	-21	-23	-23.4
**Multilayer**	**A-AuNU**	-17.8	-20.4	-19.6
	**C-AuNU**	-19.4	-22.5	-23.2
	**C_{iso}-AuNU**	-17.2	-17.8	-16.7
	**T-AuNU**	-18.2	-22.1	-23.4
	**G-AuNU**	-19.9	-19.7	-20.2
**Submonolayer**	**AG-AuNU**	-16.5	-16.1	-14.6
	**CT-AuNU**	-24.0	-21.1	-16.9
	**C_{iso}C-AuNU**	-22.8	-21.0	-21.8
	**1C9A-AuNU**	-25.8	2.48	-52.9
	**9C1A-AuNU**	-33.4	-19.2	-51.3
	**9ACTG-AuNU**	1.18	-18.8	-32.7

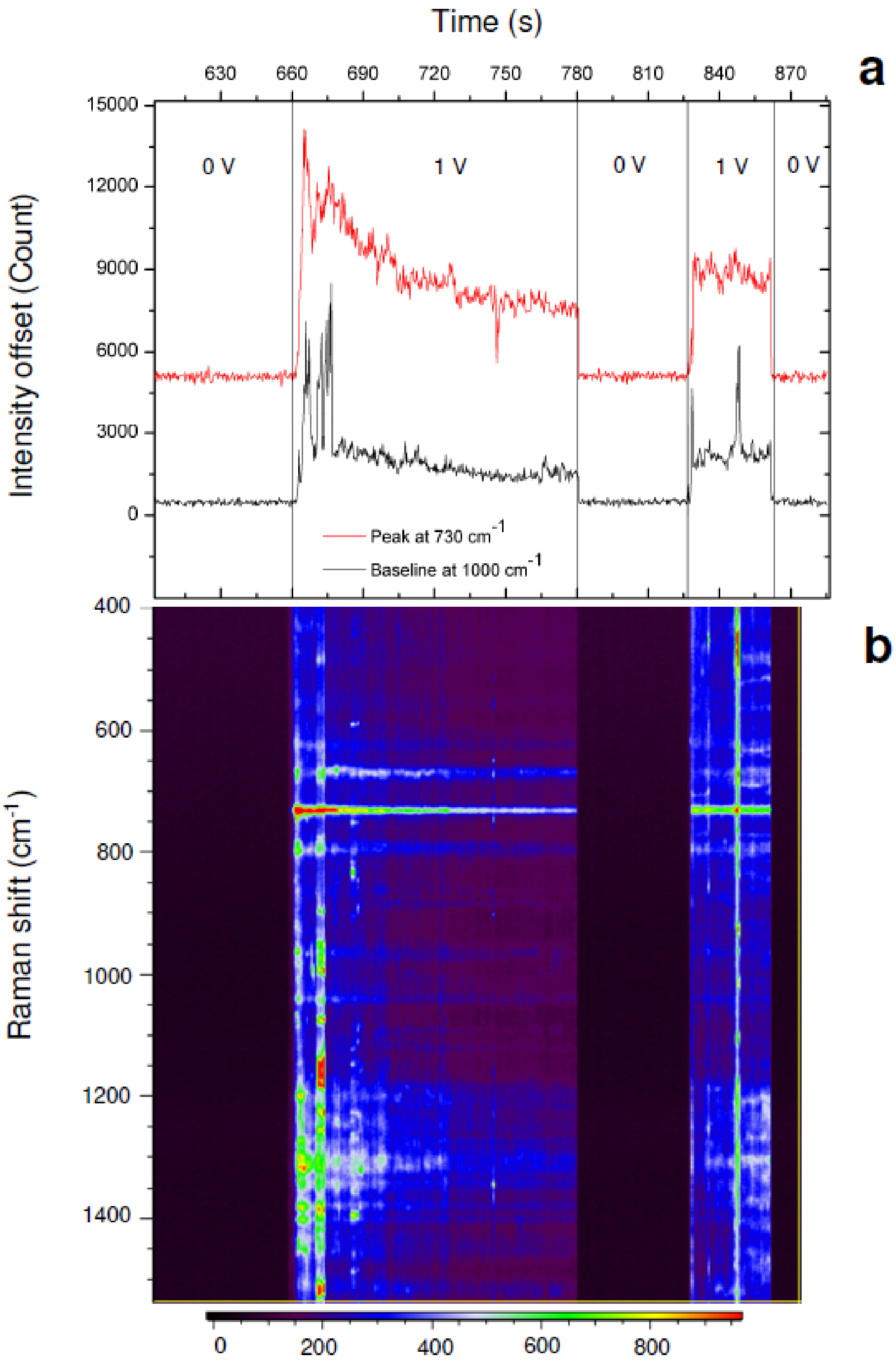

Supplementary Figure 5. Trapping dynamics of Adenine-multilayer-adsorbed gold nanourchins (A-AuNUs). (a) SERS Time trace of A-AuNU trapped and released in a nanopore by 1V bias and laser power 12 mW. (b) SERS Time series produced by the trapped A-AuNU. At 660s, trapped AuNUs produced irreproducible SERS signals as well as fluctuating baseline until 680s, which can be because there is >1 AuNUs in the nanohole. After 680s, the baseline became stable, suggesting only one AuNU was trapped in the nanohole.

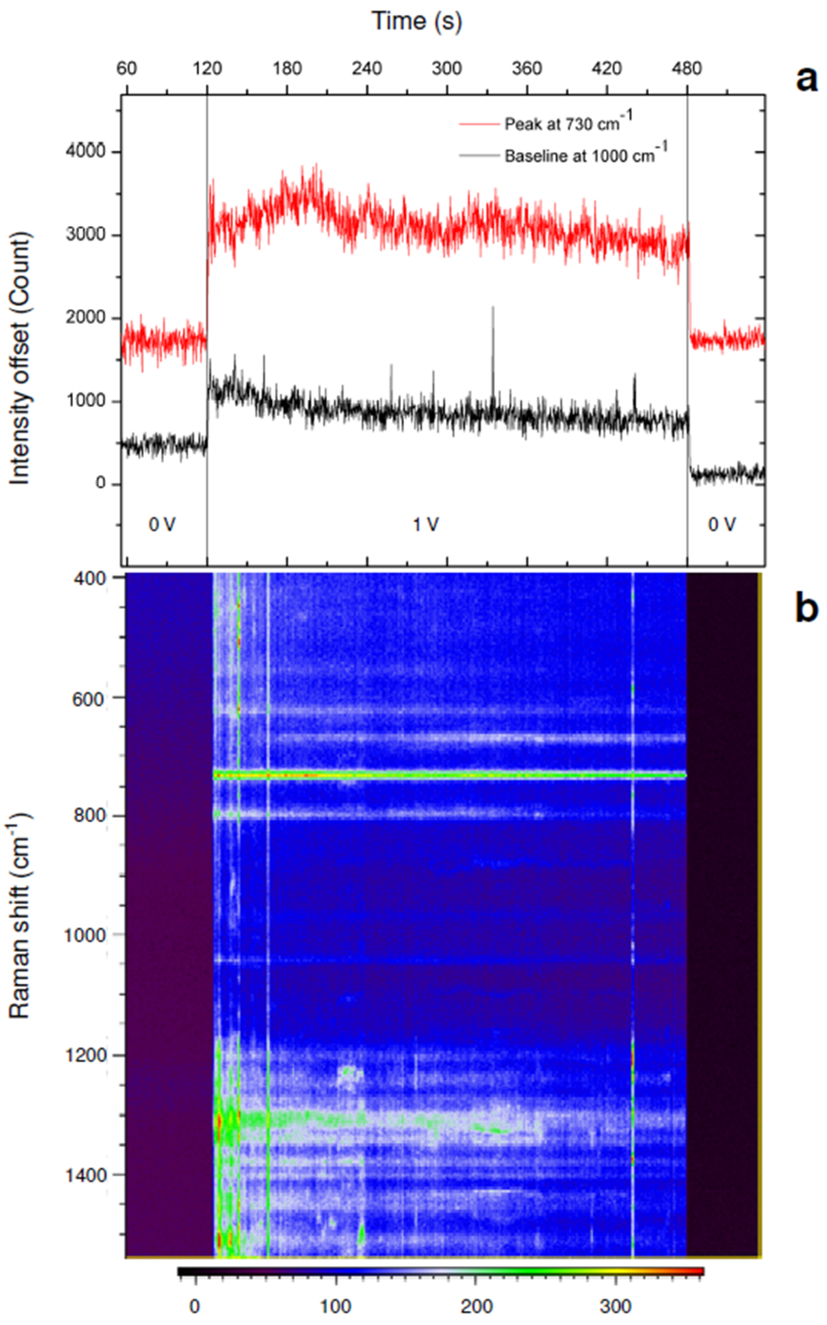

Supplementary Figure 6. Reproducible SERS spectra of a trapped Adenine-multilayer-adsorbed gold nanourchins (A-AuNUs) with a relative standard deviation (RSD) around 13%. (a) Time trace of Raman peak at 730 cm^{-1} (red) and baseline at 1000 cm^{-1} (black) of A-AuNU trapped in a nanopore by 1V bias and laser power 12 mW for 6 minutes until the bias was turned off. (b) SERS time series produced by the trapped A-AuNU.

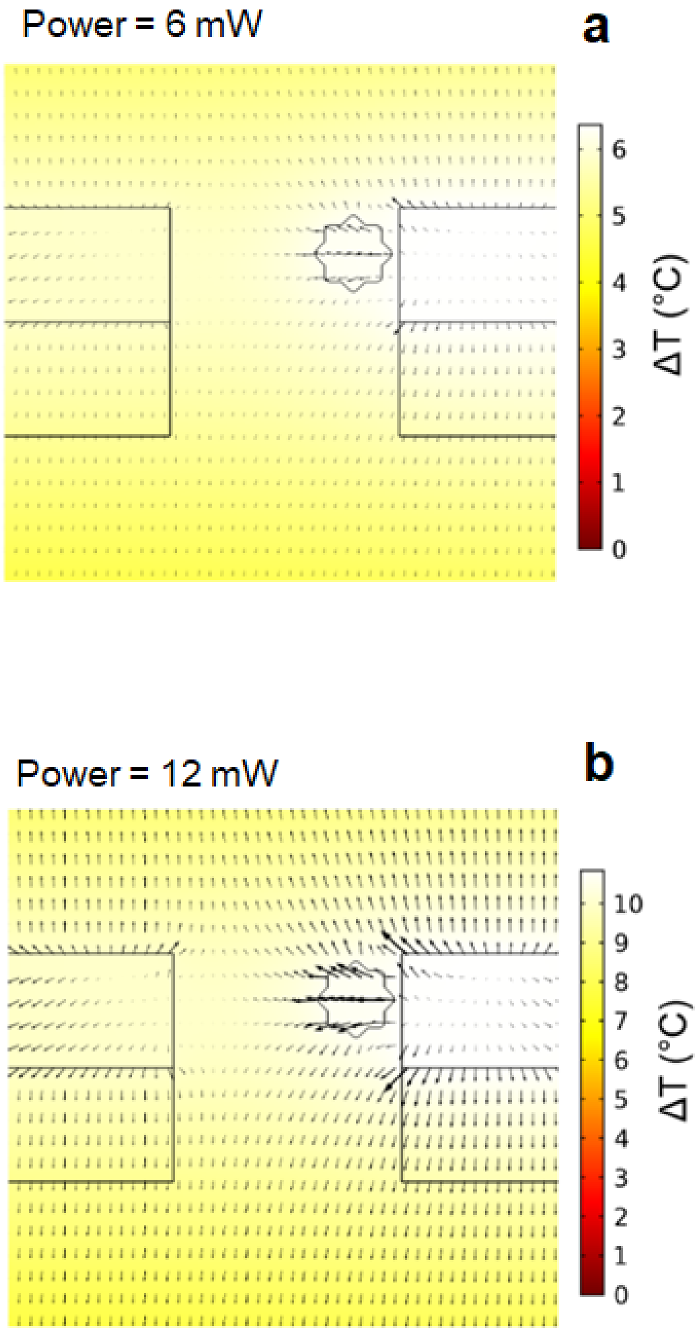

Supplementary Figure 7. The temperature rising maps for different laser powers of (a) 6 mW and (b) 12 mW. Color bars indicate the temperature rise upon laser illumination. The direction of the arrows indicates the thermal flux direction and the length of the arrows indicates the relative magnitude. Therefore, thermal flux to repel the AuNU is stronger at higher laser power.

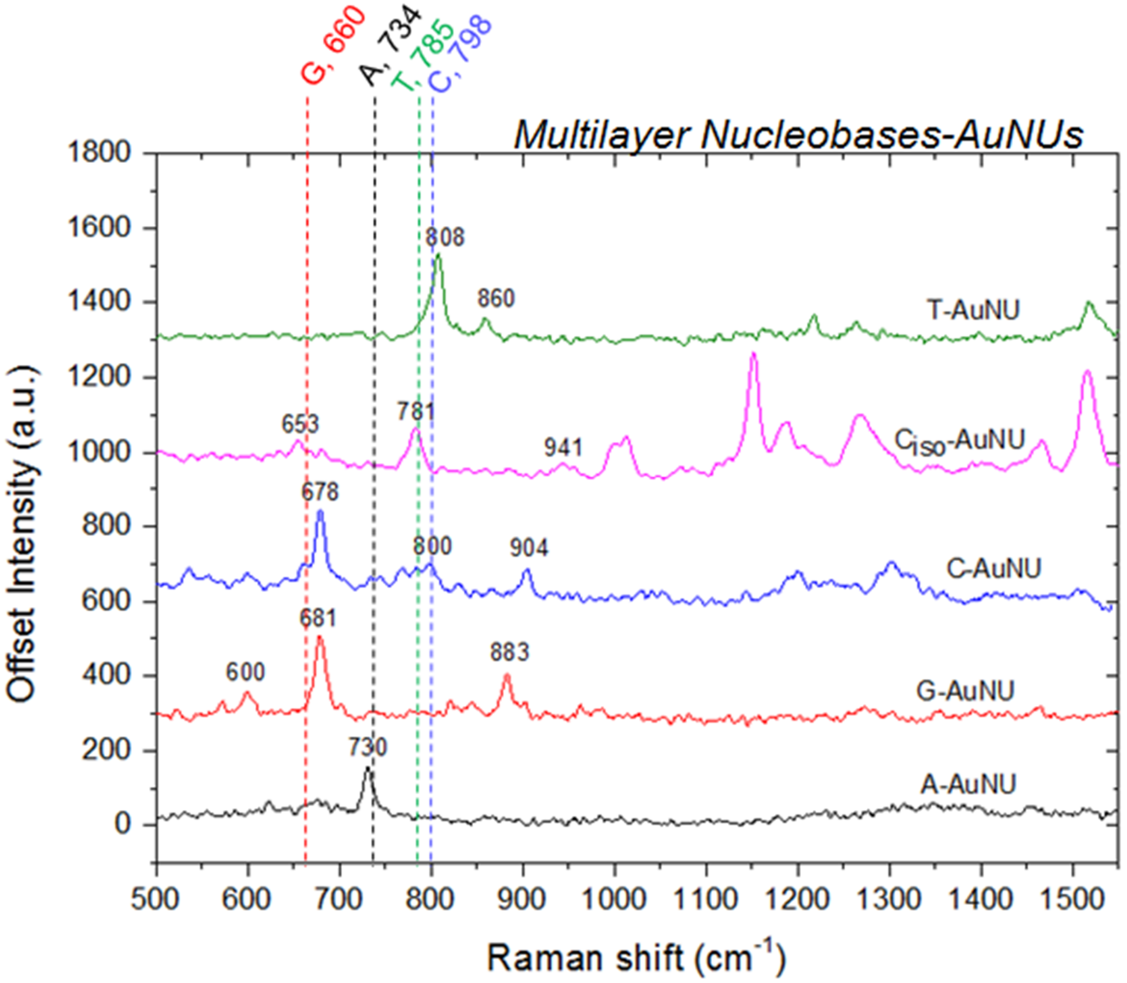

Supplementary Figure 8. SERS spectra of multilayer nucleobase-AuNUs trapped in the nanohole under different trapping conditions. The conditions are A-AuNU (1V, 6 mW), C-AuNU (1V, 12 mW), C_{iso}-AuNU (1.2V, 12 mW), G-AuNU (2V, 6 mW) and T-AuNU (4V, 6 mW), respectively. The colored dotted lines indicate the band positions of the strong ring breathing modes of each multilayer-nucleobases enhanced by silver nanoparticles without DC electric field from reference No.[3]

Supplementary Table 2. Molecular structures and assignment of the SERS bands of the multilayer nucleobase-AuNU in Supplementary Figure 8 to specific vibrational modes of the nucleobases.

Nucleobase	Molecular structure	Raman mode (cm^{-1})	Assignment[3, 4, 5]
**A**	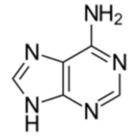	623	In-plane: 6-ring deformation
		730	In-plane: ring breathing
**C**	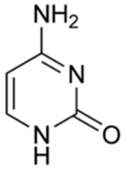	678	Out-of-plane: wag N_8-H
		800	In-plane: ring breathing
		904	Out-of-plane: asymmetric C_5-H, C_6-H wagging
**C_{iso}**	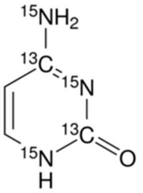	653	Out-of-plane: wag N_8-H
		781	In-plane: ring breathing
		941	Out-of-plane: asymmetric C_5-H, C_6-H wagging
**G**	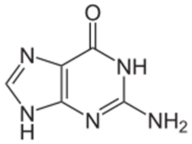	681	In-/Out-of-plane: 6-ring breathing, 5-ring deformation, wagging NH_2
		883	In-/Out-of-plane: 5-ring deformation, 6-ring deformation, wagging N_9-H, N_1-H
**T**	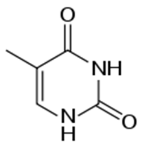	808	In-plane: ring breathing
		860	In-plane: ring deformation

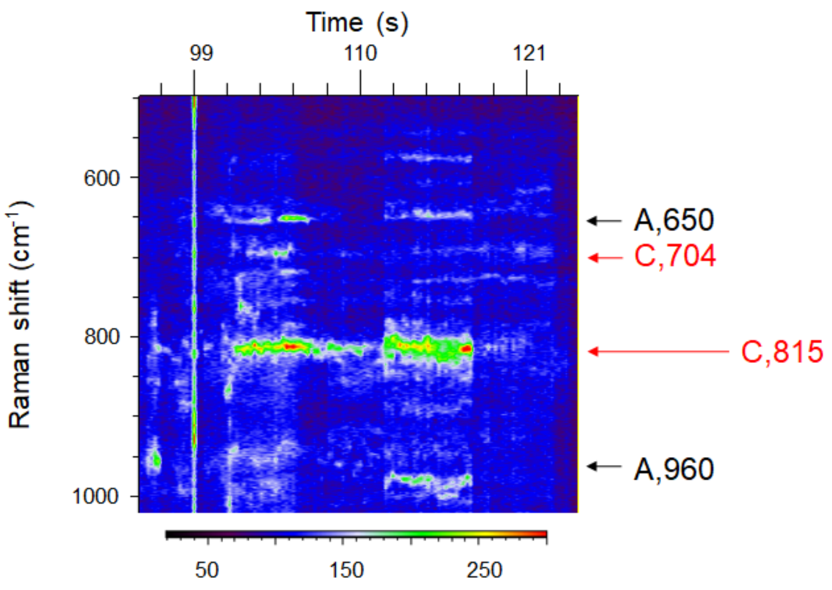

Supplementary Figure 9. SERS discrimination of single nucleobases in 5'-C AAA AAA AAA-3' oligonucleotide sub-monolayer adsorbed on the gold nanourchin (1C9A-AuNU). The color bars represent the peak intensity. The arrows indicate the frequency positions of the Raman peaks assigned to either A (black) or C (red).[3, 5] Single-C events from 107.8 to 112.2 s are indicated by the long red arrows in the SERS Time series that are extracted from 1050 SERS spectra.

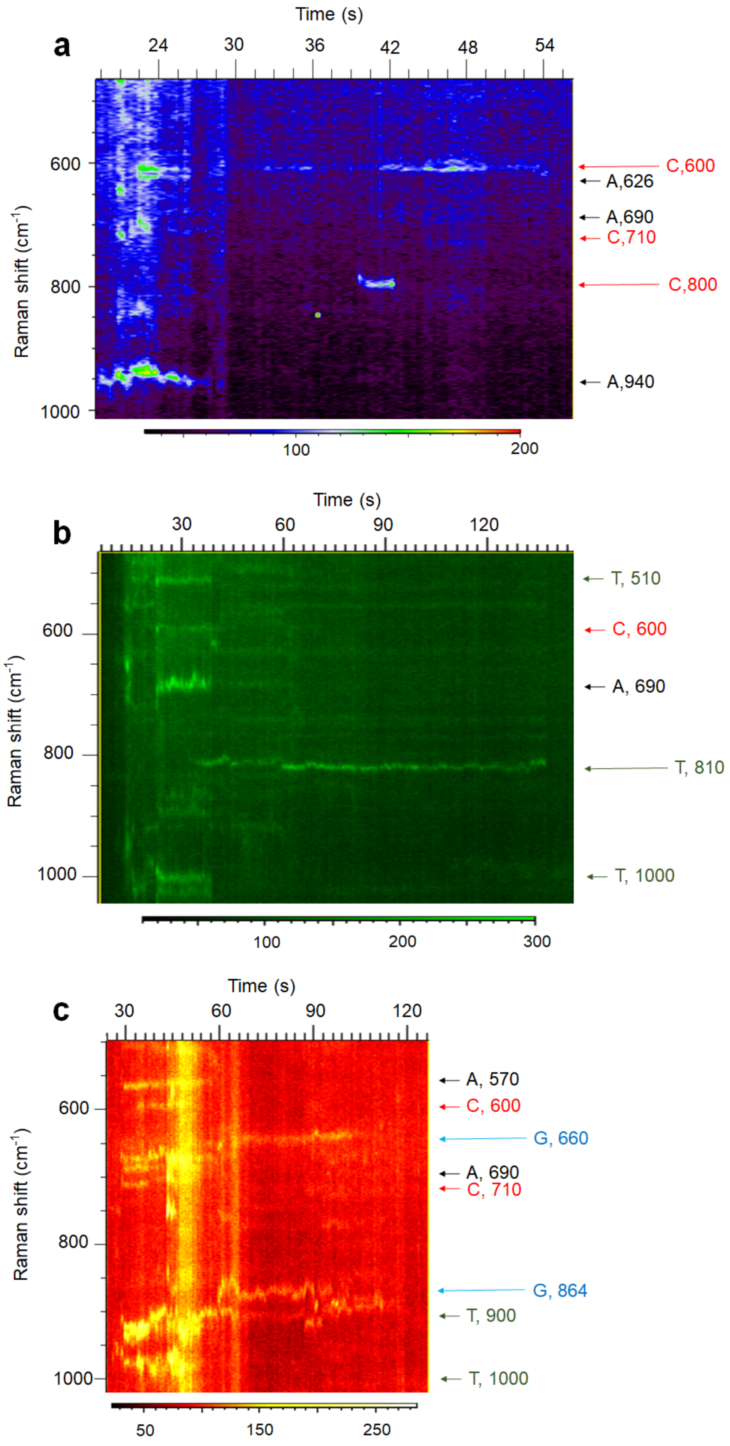

Supplementary Figure 10. Single-base discrimination in 5'-AAA AAA AAA CTG-3' oligonucleotide sub-monolayer adsorbed on the gold nanourchins (9ACTG-AuNUs). SERS Time series of (a) single Cytosine, (b) single Thymine, and (c) single Guanine are demonstrated that are extracted from 1400 spectra, respectively. The color bars represent the peak intensity. The arrows indicate the frequency positions of the Raman peaks assigned to either A (black), C (red), T (green) or G (blue).[3, 5] Single-base events are indicated by the long color arrows.

References

1. Ashkin A, Dziedzic JM, Bjorkholm JE, Chu S. OBSERVATION OF A SINGLE-BEAM GRADIENT FORCE OPTICAL TRAP FOR DIELECTRIC PARTICLES. *Optics Letters* **11**, 288-290 (1986).

2. Harada Y, Asakura T. Radiation forces on a dielectric sphere in the Rayleigh scattering regime. *Optics Communications* **124**, 529-541 (1996).

3. Madzharova F, Heiner Z, Guehlke M, Kneipp J. Surface-Enhanced Hyper-Raman Spectra of Adenine, Guanine, Cytosine, Thymine, and Uracil. *Journal of Physical Chemistry C* **120**, 15415-15423 (2016).

4. Cho KH, Joo SW. Tautomerism of cytosine on silver, gold, and copper: Raman spectroscopy and density functional theory calculation study. *Bulletin of the Korean Chemical Society* **29**, 69-75 (2008).

5. Otto C, Vandentweel TJJ, Demul FFM, Greve J. SURFACE-ENHANCED RAMAN-SPECTROSCOPY OF DNA BASES. *Journal of Raman Spectroscopy* **17**, 289-298 (1986).
