# Supplementary material for: SERS discrimination of single DNA bases in single oligonucleotides by electro-plasmonic trapping
Source: Nat Commun. 2019 Nov 22;10:5321. doi: 10.1038/s41467-019-13242-x (PMC6874578; doi:10.1038/s41467-019-13242-x)
Supplement: Supplementary file 3 — Reporting Summary [file 41467_2019_13242_MOESM3_ESM.pdf]

naturereserach

Corresponding author(s): Francesco De Angelis

Last updated by author(s): Aug 6, 2019

Reporting Summary

Nature Research wishes to improve the reproducibility of the work that we publish. This form provides structure for consistency and transparency in reporting. For further information on Nature Research policies, see Authors & Referees and the Editorial Policy Checklist .

Statistics

For all statistical analyses, confirm that the following items are present in the figure legend, table legend, main text, or Methods section.

n/a	Confirmed	
☐	☒	The exact sample size (*n*) for each experimental group/condition, given as a discrete number and unit of measurement
☐	☒	A statement on whether measurements were taken from distinct samples or whether the same sample was measured repeatedly
☒	☐	The statistical test(s) used AND whether they are one- or two-sided<br>*Only common tests should be described solely by name; describe more complex techniques in the Methods section.*
☐	☒	A description of all covariates tested
☒	☐	A description of any assumptions or corrections, such as tests of normality and adjustment for multiple comparisons
☐	☒	A full description of the statistical parameters including central tendency (e.g. means) or other basic estimates (e.g. regression coefficient) AND variation (e.g. standard deviation) or associated estimates of uncertainty (e.g. confidence intervals)
☒	☐	For null hypothesis testing, the test statistic (e.g. *F*, *t*, *r*) with confidence intervals, effect sizes, degrees of freedom and *P* value noted<br>*Give P values as exact values whenever suitable.*
☒	☐	For Bayesian analysis, information on the choice of priors and Markov chain Monte Carlo settings
☒	☐	For hierarchical and complex designs, identification of the appropriate level for tests and full reporting of outcomes
☒	☐	Estimates of effect sizes (e.g. Cohen's *d*, Pearson's *r*), indicating how they were calculated

Our web collection on statistics for biologists contains articles on many of the points above.

Software and code

Policy information about availability of computer code

Data collection	COMSOL Multiphysics® v. 5.3a, Python, Renishaw WiRE 3.4
Data analysis	COMSOL Multiphysics® v. 5.3a, Python, Renishaw WiRE 3.4, Origin 2019b

For manuscripts utilizing custom algorithms or software that are central to the research but not yet described in published literature, software must be made available to editors/reviewers. We strongly encourage code deposition in a community repository (e.g. GitHub). See the Nature Research guidelines for submitting code & software for further information.

Data

Policy information about availability of data

All manuscripts must include a data availability statement. This statement should provide the following information, where applicable:

- Accession codes, unique identifiers, or web links for publicly available datasets
- A list of figures that have associated raw data
- A description of any restrictions on data availability

More figures and analysis are available in the supplementary information. The data that support the findings of this study are available from the corresponding authors upon reasonable request.

Field-specific reporting

Please select the one below that is the best fit for your research. If you are not sure, read the appropriate sections before making your selection.

☐ Life sciences ☐ Behavioural & social sciences ☒ Ecological, evolutionary & environmental sciences

Ecological, evolutionary & environmental sciences study design

All studies must disclose on these points even when the disclosure is negative.

Study description	We used a electroplasmonic system to collect Raman spectra of nucleotide submonolayers adsorbed on gold nanoparticles, which were proved as single-molecule spectra by bi-analyte SERS (BiASERS) analysis of more than 1000 spectra of respective nucleotides.
Research sample	Nanoholes were made on a gold film deposited on SiN substrate. They had diameters of 200 nm due to plasmonic resonance at around 780 nm wavelength. The as-made substrate was then encapsulated in a PDMS chamber. The nucleotides were mixed with the gold nanoparticles for 48 hours to allow adsorption and then the gold nanoparitcles were driven by electrica bias to the gold nanohole upon 785 nm laser illumination. The diameter of the gold nanoparticles were chosen as 50 nm to reach stable trapping.
Sampling strategy	SERS spectra can be affected by many parameters and thus individual single-molecule spectra were not convincing. To unequivocally prove single-molecule detection by the BiASERS analysis, at least 1000 SERS spectra were needed for each combination of the 2 types of nucleotides to demonstrate that the single-molecule spectra were more than many-molecule spectra.
Data collection	The PDMS chamber had a cis- and a trans- chamber. The gold nanoparticles were put into the cis chamber while solvent was put in the trans chamber. By 785 nm laser illuminating one gold nanohole by a water-immersion objective, Raman spectra were collected continuously for around 5 minutes to produce around 1060 spectra by a Renishaw Raman spectrometer. During the process, electrical bias was applied between the 2 chambers to either drive the nanoparticle to the nanohole or facilitate nanoparticle trapping.
Timing and spatial scale	We started the data collection on 27 Nov, 2017 and ended on 27 Mar, 2019. During the period, we optimized the trapping and SERS signals by changing sizes of either the nanoholes and the nanoparticles.
Data exclusions	We treated the 5-mintue data from one nanohole as one unit of data for the BiASERS and excluded the unit of data that shown fluctuating spectra in both baseline and Raman peaks, because such data indicated that the nanohole was clogged with many nanoparticles.
Reproducibility	The 5-minute data can be plotted by the Renishaw WiRE 3.4 software as a contour map (SERS time series) that displaied Raman peaks with time. Only data with reproducible and stable Raman peaks in the contour map were used for the BiASERS analysis and ensure data reproducibility.
Randomization	The nucleotide submonolayer adsorption on the gold nanoparticles were by direct adsorption, which were random and not controlled by us. The nanoparticles driven to and trapped in the nanoholes to produce the SERS spectra were also random.
Blinding	The SERS measurement of the oligonucleotide, 9ACTG, was a blinding test because we could not predict which of the single-C, single-T or single-G spectra would be detected.

Did the study involve field work? ☐ Yes ☒ No

Reporting for specific materials, systems and methods

We require information from authors about some types of materials, experimental systems and methods used in many studies. Here, indicate whether each material, system or method listed is relevant to your study. If you are not sure if a list item applies to your research, read the appropriate section before selecting a response.

Materials & experimental systems

n/a	Involved in the study
☒	☐ Antibodies
☒	☐ Eukaryotic cell lines
☒	☐ Palaeontology
☒	☐ Animals and other organisms
☒	☐ Human research participants
☒	☐ Clinical data

Methods

n/a	Involved in the study
☒	☐ ChIP-seq
☒	☐ Flow cytometry
☒	☐ MRI-based neuroimaging
